# Clinical-scale 10-day TCR-T cell manufacturing using IL-2/7/15 and TGF-β promotes early memory and tissue-resident-like phenotypes and robust antitumor activity *in vitro*

**DOI:** 10.3389/fimmu.2026.1847411

**Published:** 2026-05-15

**Authors:** Yi-Ping Shih, Stephan Drokin, Olivia Burke, Huayu Huang, Amy Leung, Marco Bravo-Manriquez, Myungkyu Jang, Marina S. Syrkina, Laura Julian, Nelson Sanjuan, Eric Tran

**Affiliations:** Earle A. Chiles Research Institute, a division of Providence Cancer Institute, Portland, OR, United States

**Keywords:** adoptive cell therapy, central memory T cells, KRAS, solid cancers, stem-like T cells, TCR-gene therapy, tissue resident memory T cells

## Abstract

**Background:**

Adoptive cell therapy (ACT) using TCR-engineered T (TCR-T) cells is a promising strategy for treating solid tumors. One factor that influences the efficacy of ACT is the type of T cells used, with T cells displaying younger, less differentiated or tissue resident phenotypes associated with greater antitumor activity. We aimed to develop a rapid, clinical-scale protocol to generate younger and more potent TCR-T cells for therapy.

**Methods:**

Patient-derived PBMC were stimulated, CD8+ enriched, retrovirally transduced to express KRAS G12D-targeting TCRs, and expanded for 10 days in the presence of a novel cytokine cocktail (CKT) containing IL-2, IL-7, IL-15, and TGF-β. The impact of CKT on the phenotype, effector function, and *in vitro* antitumor activity was evaluated and compared to TCR-T cells manufactured with IL-2. This process was then adapted for clinical-scale manufacturing.

**Results:**

TCR-T cells generated with CKT displayed an increased frequency of early memory (Tn/scm) and tissue-resident (Trm)-like T cells with decreased KLRG1 expression compared to IL-2 manufactured TCR-T cells. CKT manufactured TCR-T cells demonstrated higher 4-1BB upregulation, IFN-γ, TNF, and granzyme B (GZMB) production, and enhanced killing of pancreatic and colorectal cancer cell lines in 2D and 3D tumor spheroid co-culture. Clinical-scale engineering runs yielded 3.30 and 6.15 x 10^9^ total cells that displayed similar phenotypic and functional attributes observed in the small-scale studies.

**Conclusion:**

Our novel 10-day TCR-T manufacturing protocol using IL-2, IL-7, IL-15, and TGF-β generates TCR-T cells characterized by distinct memory and tissue residency markers such as CCR7, CD103, and CD49a, and potent effector functions with the potential to improve the efficacy of adoptive cell therapy.

## Introduction

T-cell receptor-engineered T-cell (TCR-T) therapy is a type of adoptive cell therapy where a patient’s autologous peripheral blood T cells are genetically engineered to express a TCR that targets an antigen expressed by the patient’s tumor. Promising clinical activity in solid tumors has been observed with TCR-T therapy targeting tumor antigens such as NY-ESO-1 (1–7), MAGE-A3 (8, 9), MAGE-A4 (10, 11), PRAME (12), HPV16-E6 (13) and E7 (14), TP53 R175H (15), KRAS G12D (16), as well as personalized mutations (17). Approximately 40% of patients with synovial sarcoma experienced a clinical response after TCR-T therapy targeting MAGE-A4 (10), leading to the FDA approval of the first TCR-T therapy (afamitresgene autoleucel) in 2024 for this indication. However, despite promising clinical activity, there currently are no published reports of durable complete responses mediated by TCR-T therapy in patients with metastatic solid cancers outside of melanoma. Thus, there is an urgent need to improve the efficacy of TCR-T therapy for metastatic epithelial cancers.

Different T-cell subsets have different capacities to eliminate tumors. A wealth of data in mouse tumor models have demonstrated that the adoptive transfer of less differentiated CD8+ T cells such as stem cell memory (Tscm) and central memory (Tcm) T cells is more effective at mediating tumor regression than effector (Teff) and terminally differentiated effector (Tte) T cells (reviewed in (18)). Correlative data in human adoptive tumor-infiltrating lymphocyte (TIL) therapy (19) and chimeric antigen-receptor (CAR) T-cell therapy (20–24) also have implicated a role for less differentiated T cells in mediating antitumor responses. In addition to Tscm and Tcm, other T-cell subsets may be desirable for use in adoptive cell therapy (ACT). For example, tissue resident memory T cells (Trm) are associated with favorable outcomes and clinical responses to immunotherapy in a variety of human cancers (reviewed in (25) and (26)). While there are limited studies on the use of Trm for ACT, the transfer of Trm-like CAR-T cells was superior to conventional CAR-T cells in solid and liquid tumor xenograft models (27), and the transfer of T cells resembling Trm through deletion of *Vhl* or overexpression of the Runx3 transcription factor led to improved control of syngeneic mouse tumors (28, 29). Clinically, we previously reported a patient with metastatic pancreatic ductal adenocarcinoma (PDAC) who achieved a clinical response following the infusion of KRAS G12D-targeted TCR-T cells; these cells were skewed toward a Trm-like phenotype by manufacturing in the presence of TGF-β (16).

A unique feature of ACT is the ability to manipulate cell culture conditions to generate T cells with desired phenotypes and functional attributes. Indeed, cytokines such as IL-7, IL-15, and IL-21 can promote the generation of human Tscm and/or Tcm (30–34) while TGF-β has been used to generate human Trm-like T cells (16, 27). Here, we developed a 10-day TCR-T cell manufacture protocol that uses the novel cytokine combination of IL-2, IL-7, IL-15, and TGF-β and G-Rex bioreactors. We found that TCR-T products manufactured with this cytokine cocktail (CKT) were enriched in Tscm, Tcm, and Trm-like cells compared to cells manufactured with IL-2 alone. Moreover, CKT manufactured TCR-T cells exhibited superior cytotoxic ability and effector cytokine production against tumor cell lines than those grown with IL-2 alone. Engineering runs in our cleanroom facility using patient peripheral blood mononuclear cells (PBMCs) produced 3.3–6.1 × 10^9^ CD8-enriched cells with an average TCR transduction efficiency of 85%. Thus, this new manufacturing process can generate clinically relevant numbers of TCR-T cells with desirable attributes for ACT.

## Materials and methods

### Patient samples

Leukapheresis products were obtained from patients through an IRB-approved tissue procurement protocol (IRB number 2018000418). PBMCs were isolated from leukapheresis products using standard Ficoll-Paque density gradient methods and PBMCs were cryopreserved in CryoStor10 (BioLife Solutions). PBMCs used in this study were derived from two patients with colorectal cancer (CRC), CRI-5268 and CRI-5126 (both HLA-C*08:02 genotype), and two patients with pancreatic ductal adenocarcinoma (PDAC), CRI-5178 and CRI-2890 (HLA-A*11:01 and HLA-C*08:02 HLA genotype, respectively).

### Cell lines

The endogenous KRAS G12D-positive cell lines including the pancreatic adenocarcinoma cell lines HPAC and HPAFII as well as the colorectal adenocarcinoma cell lines LS513 and LS180 were acquired from the American Type Culture Collection (ATCC). These cell lines were retrovirally transduced to stably express HLA-C*08:02 and green fluorescent protein (GFP), or HLA-A*11:01 and GFP, with the HLA and GFP genes separated by a 2A self-cleaving peptide sequence. All cell lines were cultured in Dulbecco’s Modified Eagle Medium (DMEM, Gibco) supplemented with high glucose, GlutaMax, 10% fetal bovine serum (FBS, Gibco), and 1% penicillin/streptomycin.

### TCRs and retroviral vectors

The three KRAS G12D-reactive TCRs used in this study have been previously characterized (16, 35, 36). Two HLA-C*08:02-restricted TCRs were used: the C08-9mer TCR which recognizes the 9mer GA**D**GVGKSA peptide, and the C08-10mer TCR which recognizes the 10mer GA**D**GVGKSAL peptide. The third TCR, A11-10mer TCR, is HLA-A*11:01 restricted and recognizes the 10mer VVVGA**D**GVGK peptide. Each TCR contains the mouse TCRα and TCRβ constant regions, which promotes pairing of the introduced TCR and allows for the identification of the TCR-transduced T cells using an anti-mouse TCRβ constant region antibody. Gammaretroviral vector for each TCR was produced under GMP by the Vector Production Facility at Cincinnati Children’s Hospital Medical Center. Retroviral vectors (RV) passed safety tests including cell-based assays for adventitious viruses and replication competent retrovirus, as well as mycoplasma, sterility, and endotoxin. Adventitious virus and replication competent retrovirus testing on the RV was performed by the National Gene Vector Biorepository at Indiana University which is funded through the National Heart Lung and Blood Institute contract 75N92019D00018.

### TCR-T cell transduction and expansion, small-scale

PBMC were thawed and 48 × 10^6^ cells were stimulated with 50 ng/mL anti-CD3 (clone OKT3, Miltenyi Biotec) in TCR-CKT2–5 complete medium which comprised AIM-V CTS media (Gibco), 5% human AB serum (GeminiBio) with the appropriate cytokines 300 IU/mL IL-2 (R&D Systems, GMP-grade), 30 ng/mL IL-7 (R&D Systems, GMP-grade), 20 ng/mL IL-15 (R&D Systems, GMP-grade), and 5 ng/mL TGF-β (Biolegend, GMP-grade). PBMC were seeded in one T-75 flask (48 mL/flask, 1 × 10^6^/mL) and incubated at 37 °C, 5% CO_2_ for two days. Non-treated tissue culture 6-well plate was coated with 10 µg/mL RetroNectin (2 mL/well, Takara Bio) a day prior to transduction and stored overnight at 4 °C. After two days, stimulated cells were harvested and CD8^+^ T cells were enriched by CD4^+^ depletion using CliniMACS CD4 MicroBeads at 3 times the recommended bead amount and LS columns (Miltenyi Biotec). RetroNectin plates were loaded with 4 mL retroviral vector (optimized dilution to achieve <5 VCN/cell) and centrifuged at 2000 × *g*, 32 °C for 2 h. Retroviral supernatant were then aspirated and 2 × 10^6^ CD8^+^ T cells were added per transduced well (4 mL/well). Plates were centrifuged at 400 × *g* for 10 min and incubated overnight at 37 °C, 5% CO_2_. After one day, transduced cells from one well were transferred into a well of a G-Rex 6M-2 plate (Wilson Wolf) and filled to 20 mL using TCR-CKT2–5 media. On day 7, 16 mL of media was removed from each well, and replenished with fresh TCR-CKT2–5 media. Transduced T cells were harvested on day 10 for experiments, quality control assays, and remaining cells were cryopreserved using CryoStor10 (BioLife Solutions).

### TCR-T cell transduction and expansion, clinical-scale

Manufacturing was performed in our GMP-capable cleanroom facility. PBMCs were thawed and 1.2 × 10^9^ cells were stimulated using the same GMP-grade reagents as the small-scale process and seeded in six T-300 flasks (192 mL/flask, 1 × 10^6^/mL) and incubated at 37 °C, 5% CO_2_ for two days. Non-treated tissue culture 6-well plates were coated with 10 µg/mL RetroNectin (2 mL/well, Takara Bio, GMP-grade) a day prior to transduction and stored overnight at 4 °C. For one TCR, 12 plates (72 wells) were prepared; for two TCRs, 6 plates per TCR (36 wells each). After two days, stimulated cells were pooled and CD8^+^ T cells were enriched by CD4^+^ depletion using CliniMACS CD4 MicroBeads at 3 times the recommended bead amount and LS columns (Miltenyi Biotec). RetroNectin plates were loaded with 4 mL retroviral vector (optimized dilution to achieve <5 VCN/cell) and centrifuged at 2000 × *g*, 32 °C for 2 h. Retroviral supernatants then were aspirated and 2 × 10^6^ CD8^+^ T cells were added per retronectin-coated well (4 mL/well, 144 × 10^6^ CD8^+^ cells total). Plates were centrifuged at 400 × *g* for 10 min and incubated overnight at 37 °C, 5% CO_2_. After one day, transduced T cells were transferred into a G-Rex 100M flask (Wilson Wolf) (34 or 35 wells per flask) and filled to 1000 mL using TCR-CKT2–5 media. On day 7, 600 mL of media was removed from each flask and fresh TCR-CKT2–5 media was added to the flasks. Transduced T cells were harvested on day 10 via the LOVO system (Fresenius Kabi) and formulated into the drug product with 250 mL buffer consisting of Plasmalyte (Baxter) + 2% human serum albumin (GRIFOLS). The drug products underwent experiments, quality control assays, and aliquots of cells were cryopreserved using CryoStor10 (BioLife Solutions). Key quality control assays included transduction efficiency (mTCRβ) by flow cytometry, MSGV1 vector copy number (VCN) by QPCR, in-process testing for replication-competent retrovirus (RCR) by QPCR for the envelope RD114, sterility testing using the BacT/Alert system (bioMérieux) with iLYM, iNST, and iAST culture bottles to detect a broad range of bacteria, yeasts, and molds, mycoplasma testing using the MycoTOOL RT-PCR kit (Roche), and endotoxin detection with EndoSafe nexgen-PTS (Charles River).

### Flow cytometry staining

For T-cell phenotyping studies, unless otherwise indicated, cells were thawed and rested overnight in media containing the cytokines that were used to manufacture the TCR-T cells. The next day, cells were harvested and stained at 10 µL/well in a 96-well U-bottom plate with a pre-titrated antibody panel prepared in FACS buffer (PBS with 2% FBS, 2 mM EDTA). Cells were stained for 30 min at 4 °C in the dark, washed with FACS buffer, and then resuspended in 100 µL of FACS buffer containing propidium iodide for live/dead cell analysis. Samples were acquired with a Cytoflex LX cytometer and analyzed with FlowJo v10. The following antibodies were used (all from BioLegend, except the γδ TCR antibody which was from BD Biosciences): AF700-CD3 (SK7), BV510-CD8 (RPA-T8), PE/Cy7-CD45RO (UCHL1), BV421-CCR7 (G043H7), FITC or PE/Cy7-CD103 (Ber-ACT8), Alexa Fluor647-CD49a (TS2/7), APC-fire 750-CD69 (FN50), BV421- γδ TCR (11F2), APC-CD56 (5.1H11), PE-PD1 (EH12.2H7), PE-KLRG1 (SA231A2), APC-fire750-LAG3 (11C3C65), APC-fire 750 or PE-mTCRβ (H57-597), APC-4-1BB (4B4-1), APC-fire 750-CD4 (SK3).

### Overnight co-culture assay to evaluate T-cell activation by flow cytometry

Tumor cell lines were harvested using 2.5% trypsin-EDTA (Life Technologies). Once the cells detached from the plates, DMEM media was added to quench the reaction. Tumor cells then were harvested, spun down and resuspended in the DMEM media. 10,000 tumor cells in 100 ul were seeded per well of a 96-well flat bottom plate and left to recover and adhere overnight. The following day, T cells were harvested, washed, and then resuspended in 50/50 media without exogenous cytokines. 50/50 media comprised 50% AIM-V (Gibco) with 50% RPMI 1640 medium (Cytiva) containing 10% human AB serum (Valley Biomedical), 25mM Hepes (Cytiva), 10 µg/ml Penicillin-Streptomycin solution (Cytiva), 2 mM GlutaMax (Gibco), and 5 µg/ml Gentamicin (Thermo Fisher Scientific). 50,000 T cells in 100 µL then were added per well for co-culture. After overnight incubation, supernatants and cells were harvested and each transferred to a new 96-well U-bottom plate. Cells underwent staining with antibodies targeting CD3, CD8, mTCRβ, CD103 and 4-1BB followed by flow cytometry analysis as described above.

### Analysis of cytokines in supernatants

Co-culture assays were set up as described above with the following modifications: for coculturing with tumor cell lines, 50,000 tumor cells were plated either 4h or overnight before coculture with 100,000 T cells per well. For coculturing with peptide-pulsed antigen presenting cells (autologous PBMC), PBMC first were thawed, resuspended in 50/50 media at 4 × 10^6^ cells/mL and rested overnight in an ultra-low attachment flask at 37 °C and 5 % CO_2_. The following day, the PBMC were harvested, washed, and seeded at 2 × 10^6^ cells/well (1 mL) in ultra-low attachment 24-well plates. The cells then were pulsed for 2–4 h at 37 °C with 1 µg/mL of either wild-type KRAS or mutant KRAS G12D peptides (HPLC-purified, from Genscript or Lifetein). The peptides used were GA**D**GVGKSA and GA**D**GVGKSAL for the HLA-C*08:02-restricted TCRs and VVVGA**D**GVGK for the HLA-A*11:01-restricted TCR. PBMC were then washed twice with PBS and resuspended in 50/50 media and 2 × 10^5^ PBMC were cocultured with 1 × 10^5^ T cells. After overnight coculture, 140 µL of supernatants were harvested from each well and frozen. Supernatants were thawed and the LEGENDplex™ Human CD8/NK Panel V02 (BioLegend) was used for multiplex cytokine quantification following the manufacturer’s protocol except with the modification that all experimental sample and reagent volumes were each reduced to 15 µL per well. Supernatants were not diluted for the assay. Samples and standards were acquired on a Cytek Aurora flow cytometer. Data was analyzed using the LEGENDplex™ Data Analysis Software Suite (BioLegend).

### *In vitro* cancer cell killing assay, 2D culture

Human cancer cell lines stably expressing HLA-C*08:02 and GFP were seeded into a 96-well flat bottom plate at a density of 1 × 10^4^ cells per well in DMEM media. The following day, TCR-Td cells were added to tumor cells at the indicated effector to target (E:T) ratios and cocultured for 5 days in an incubator at 37 °C with 5% CO2. GFP signal was monitored using the Cellcyte live-cell analysis system (Cytena). Images were captured every 12 hours for up to 120 hours. GFP signal intensity in each well was quantified using Cellcyte software. A decrease in GFP signal represented tumor cell death and was correlated with morphological changes in the tumor cells consistent with cell death such as cell shrinkage, membrane blebbing, and loss of adhesion.

### *In vitro* cancer cell killing assay, 3D spheroid culture

Tumor spheroids were generated using HPAC-C*08:02-GFP, HPAFII-C*08:02-GFP, LS513-A*11-GFP and LS180-A*11-GFP cell lines. For spheroid formation, 2,500–5,000 tumor cells per well were seeded into 96-well ultra-low attachment PrimeSurface plates (S-bio) in 100 μL of complete medium. Plates were centrifuged at 125 × g for 10 minutes at room temperature to facilitate cell aggregation. Spheroids were incubated at 37 °C with 5% CO_2_ for 72 hours to allow for compact spheroid formation. On the day of the assay, 100 μL of T cells were added to achieve the desired E:T ratios. Spheroid size and fluorescence intensity were quantified using Cellcyte software.

### Statistical methods

Paired t-tests were performed using GraphPad Prism version 10.6.1 (GraphPad Software, San Diego, CA, USA), with *p* < 0.05 considered statistically significant. Graphs display mean ± SEM for biological replicates, or mean ± SD for technical replicates.

## Results

### Manufacturing TCR-T cells with the cytokine cocktail IL-2, IL-7, IL-15, and TGF-β enriches for early memory (Tn/scm) and tissue-resident memory (Trm)-like phenotypes

**Figure 1:**
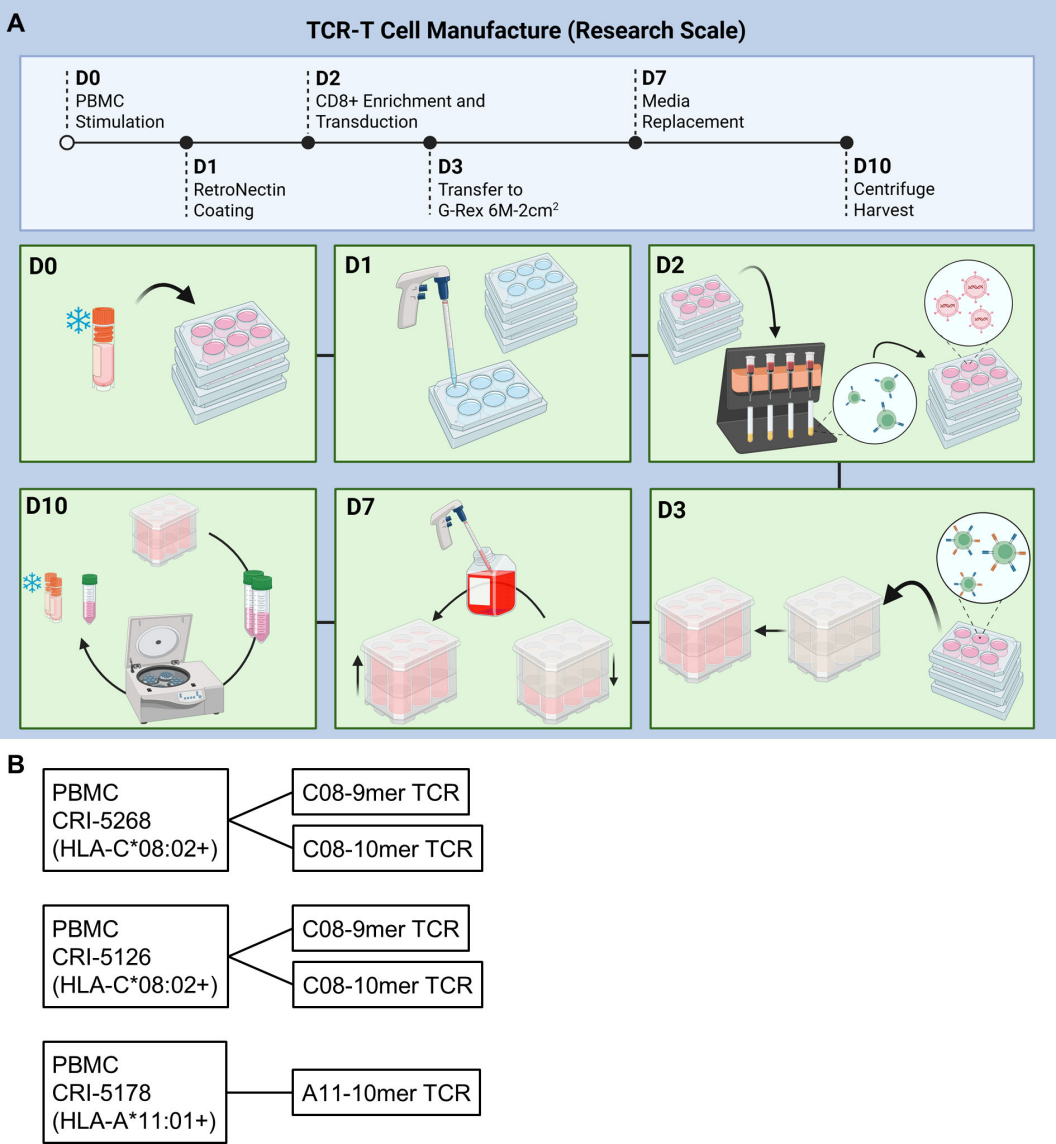
10-day TCR-T manufacture process. **(A)** Schematic and **(B)** samples evaluated and TCRs used in the small research-scale studies. Each sample was manufactured with either IL-2 or the cytokine cocktail (CKT) IL-2, IL-7, IL-15, and TGF-β. **(A)** was generated with BioRender.

**Figure 2:**
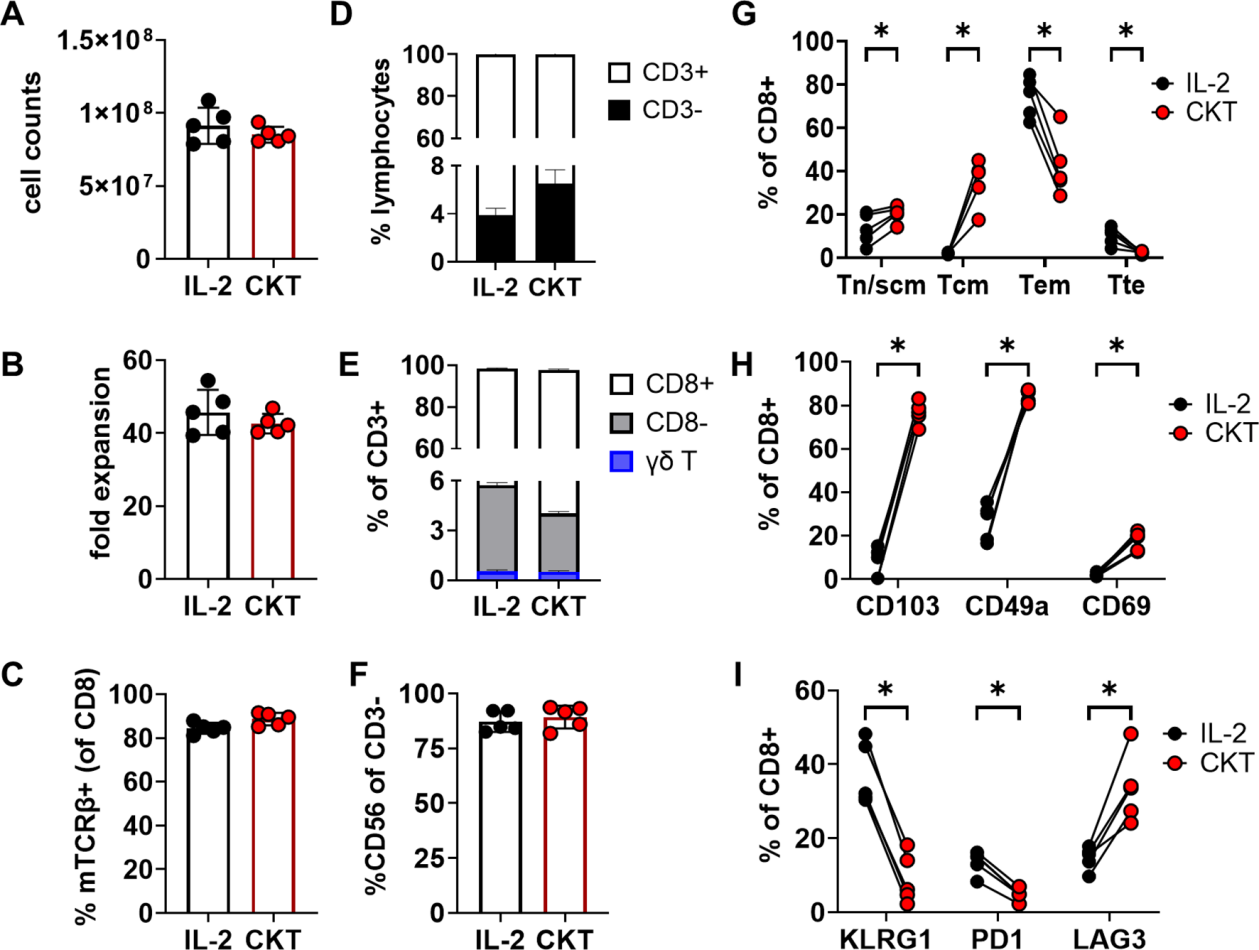
Cell number and phenotype of TCR-T cells after manufacture with IL-2 or cytokine cocktail (CKT) for 10 days. **(A)** Cell number, **(B)** fold expansion (from day 2 onwards), **(C)** transduction efficiency as determined by flow cytometric analysis of the mouse TCRβ constant region which is engineered into the TCRs. **(D)** Percent CD3+/- cells of live lymphocytes. **(E)** Frequencies of CD8+/- and γδ T cells. **(F)** Percent CD56+ cells of live CD3- cells. **(G)** T-cell differentiation, **(H)** tissue resident (Trm) and **(I)** exhaustion/activation markers were evaluated by flow cytometric analysis. Tn/scm: CD45RO-CCR7+; Tcm: CD45RO+CCR7+; Tem: CD45RO+CCR7-; Tte: CD45RO-CCR7-. Data from the three patient PBMCs and five TCR-T products are shown. *p < 0.05 by paired t-test.

Our previous TCR-T manufacturing process comprised of two *in vitro* stimulation steps that totaled nearly 4 weeks (16). Toward the goal of generating younger and more potent TCR-T cells for ACT, we shortened the manufacture time to 10 days, incorporated a CD8-enrichment step, and evaluated the use of IL-2, IL-7, IL-15, and TGF-β during cell culture (**Figure 1A**). The CD8-enrichment step was included to better standardize product composition across variable patient samples and promote optimal potency, given that HLA-I-restricted TCRs typically have higher functional avidity in CD8 T cells than in CD4 T cells. PBMC from three patients, two with colorectal cancer (CRC; CRI-5268 and CRI-5126) and one with pancreatic ductal adenocarcinoma (PDAC; CRI-5178) were thawed and stimulated with anti-CD3 (OKT3) in the presence of either IL-2 or the cytokine cocktail (CKT) IL-2, IL-7, IL-15, and TGF-β. Two days later, CD8 cells were enriched by removing CD4 cells using magnetic beads followed by genetic insertion of KRAS G12D-reactive TCRs with gammaretroviral vectors. CRI-5268 and CRI-5126 T cells were engineered to express two different HLA-C*08:02-restricted TCRs (each TCR in a separate aliquot), while CRI-5178 T cells were engineered to express an HLA-A*11:01 restricted TCR, resulting in five different samples for evaluation (**Figure 1B**). Cells were expanded for a total of 10 days prior to evaluation. As seen in **Figures 2A–C**, cell expansion and TCR transduction efficiency were comparable between the IL-2 and CKT conditions despite the presence of TGF-β in CKT. T cells comprised the majority (> 90%) of total cells (**Figure 2D**), and within this T-cell population, most were CD8+ T cells while γδ T cells accounted for less than 1% in both IL-2 and CKT manufacture groups (**Figure 2E**). CD56+ NK cells dominated the CD3-negative cell compartment (**Figure 2F**). Collectively, these results demonstrate efficient enrichment of CD8+ T cells from patient PBMCs that initially contained 65 to 80% CD4+ T cells (**Supplementary Figure 1**). Notably, TCR-T cells generated with CKT were enriched in naïve or stem cell memory (Tn/scm, CD45RO-CCR7+) and central memory (Tcm, CD45RO+CCR7+) as well as tissue resident memory-like (Trm, CD103, CD49a, and CD69) T cells compared to TCR-T generated with IL-2 (**Figures 2G, H**). The terminal differentiation marker KLRG1 and activation/exhaustion marker PD-1 were decreased while LAG3 was increased on CKT TCR-T cells compared to IL-2 TCR-T cells (**Figure 2I**). Representative flow cytometry plots are shown in **Supplementary Figure 2**.

### TCR-T cells manufactured with IL-2, IL-7, IL-15, and TGF-β display robust effector function and antitumor activity *in vitro*

**Figure 3:**
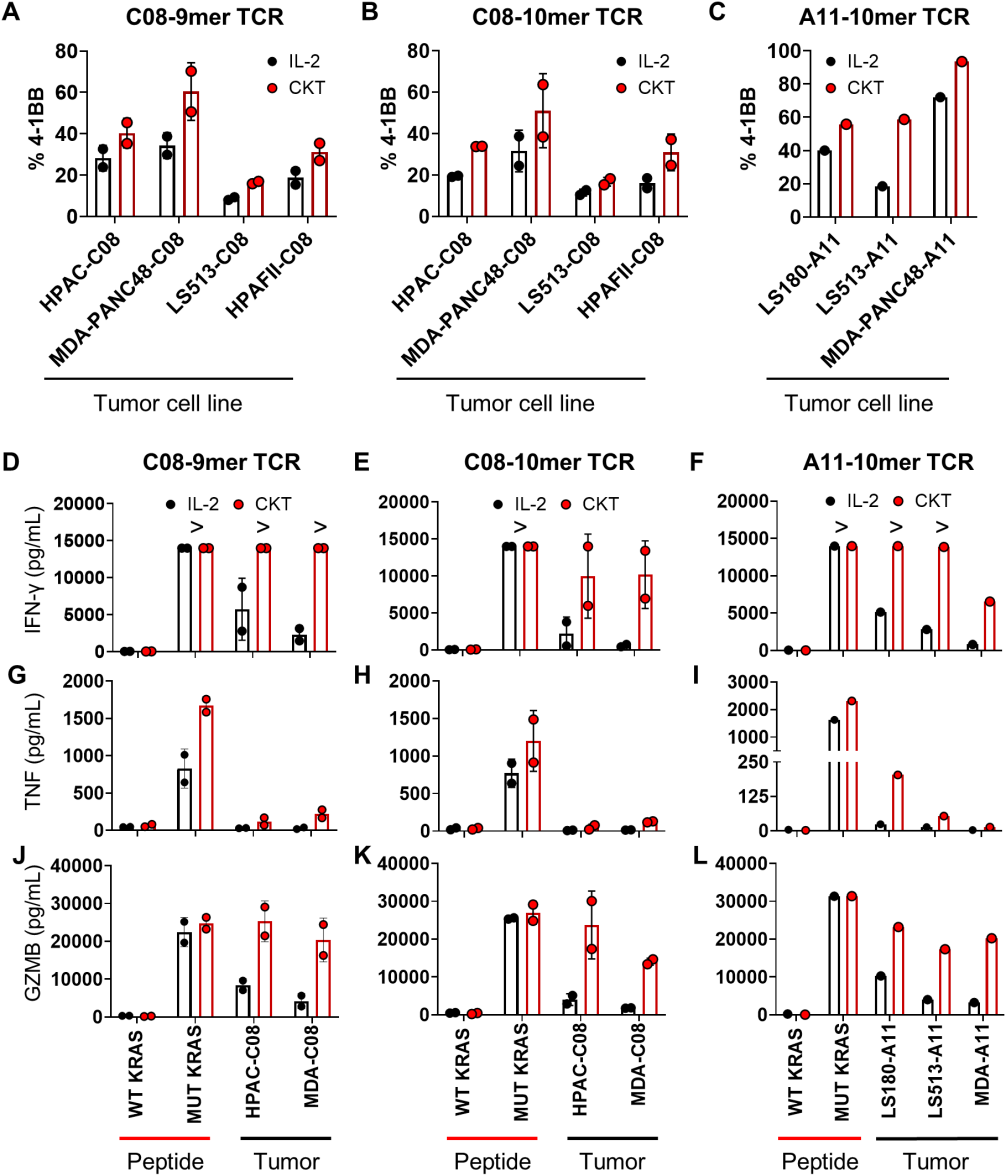
Tumor cell line reactivity and effector function of KRAS G12D-reactive TCR-T cells manufactured with either IL-2 or CKT. IL-2 (black) or CKT (red) manufactured TCR-T cells were cocultured overnight with the indicated KRAS G12D+ tumor cell lines expressing either HLA-C*08:02 (C08) or HLA-A*11:01 (A11), or autologous PBMC pulsed with 1 µg/mL of wild-type (WT) or mutated (MUT) KRAS peptide and **(A–C)** 4-1BB expression was measured by flow cytometry and **(D–F)** IFNγ, **(G–I)** TNF, and **(J–L)** granzyme B (GZMB) was measured in the supernatants using the LegendPlex assay. For the C08-9mer TCR and C08-10mer TCR, the two HLA-C*08:02+ patient samples (CRI-5126 and CRI-5268) were pooled. Flow cytometry data were gated on CD3+CD8+mTCRβ+ T cells. “>“ greater than assay detection limit. Coculture assays are representative of at least 2 independent experiments. LEGENDplex data were from one experiment with technical replicates.

**Figure 4:**
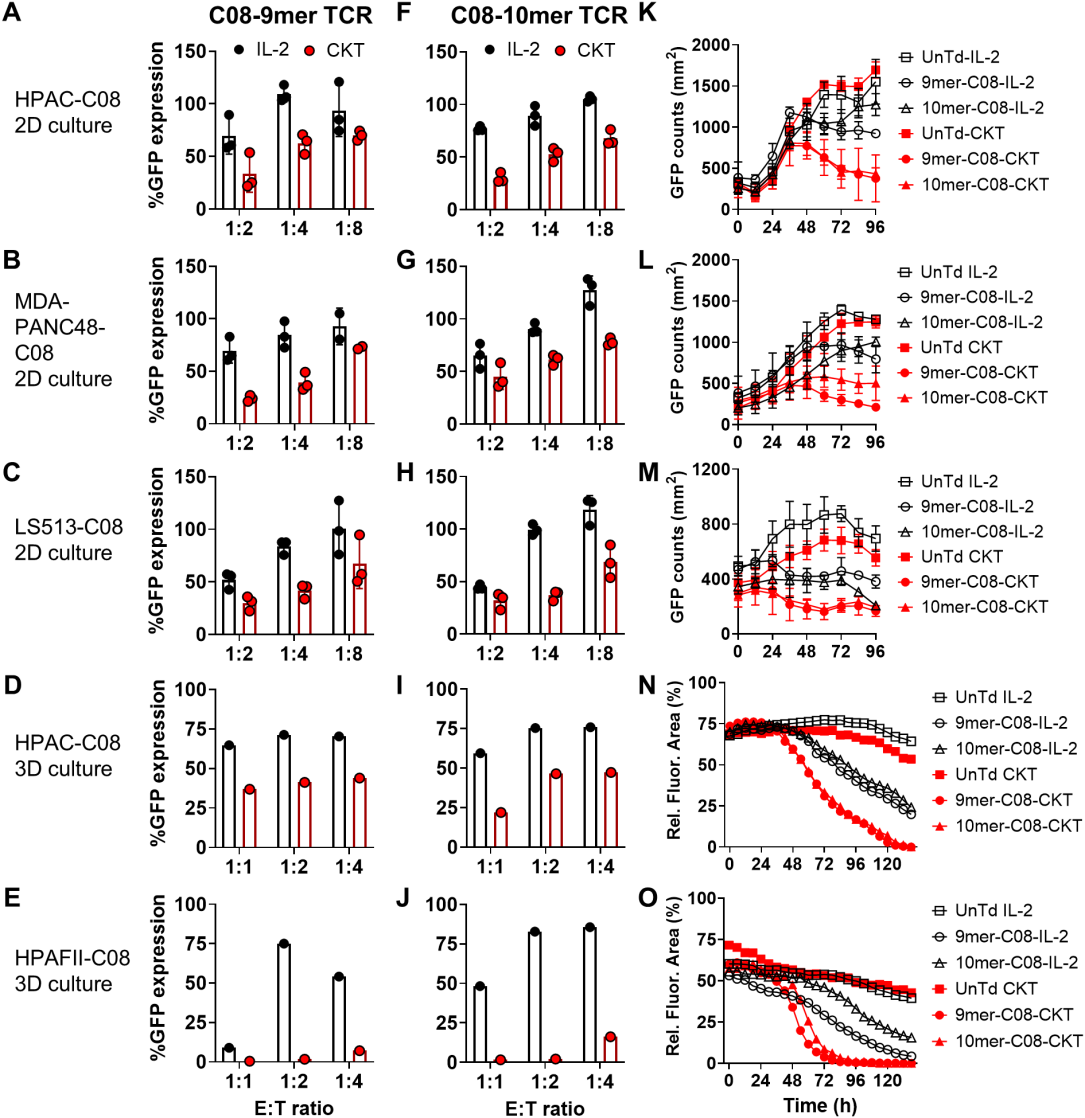
Tumor cell killing by IL-2 or CKT manufactured TCR-T cells from patient CRI-5268 in 2D and 3D coculture. **(A–E)** 9mer-C08 TCR-T cells manufactured with IL-2 (black) or CKT (red) were cocultured with the indicated KRAS G12D+ and HLA-C*08:02+ GFP-expressing tumor cell lines at various effector to target (E:T) ratios in **(A–C)** 2D or **(D, E)** 3D spheroid culture and GFP was quantitated at 72 h. **(F–J)** Same as **(A–E)** except with 10mer-C08 TCR-T cells. 100% GFP expression indicates the % GFP+ tumor cells in coculture wells containing control untransduced T cells manufactured with IL-2 or CKT. **(K–O)** IL-2 (black) or CKT (red) manufactured untransduced (UnTd), 9mer-C08, or 10mer-C08 TCR-T cells were cocultured with the same KRAS G12D+ and HLA-C*08:02+ GFP-expressing tumor cell lines described above at 1:2 E:T ratio in **(K–M)** 2D culture, or 1:4 E:T ratio in **(N, O)** 3D spheroid culture and GFP was quantitated over time using the Cellcyte live cell imager. Rel. Fluor. Area: relative fluorescence area. All data are representative of at least 2 independent experiments.

To evaluate the *in vitro* antitumor function of the TCR-T cells, we first measured expression of the T-cell activation marker 4-1BB after coculture with cancer cell lines expressing endogenous KRAS G12D that were transduced to express either HLA-C*08:02 or HLA-A*11:01. Across all three KRAS G12D TCRs and three patient donor T cells, CKT manufactured TCR-T displayed increased 4-1BB expression compared to IL-2 manufactured TCR-T cells (**Figures 3A–C**). While both IL-2 and CKT manufactured TCR-T cells could specifically produce relatively high levels of effector molecules upon stimulation with mutated KRAS peptides, CKT manufactured TCR-T cells secreted higher levels of IFN-γ (**Figures 3D–F**), TNF (**Figures 3G–I**), and granzyme B (**Figures 3J–L**) after coculture with multiple tumor cell lines compared to IL-2 manufactured TCR-T cells. This was associated with enhanced *in vitro* killing of different KRAS G12D+ GFP-expressing tumor cell lines expressing the appropriate HLA restriction element across different effector to target ratios in both 2D and 3D spheroid cultures by CKT versus IL-2 manufactured TCR-T cells as determined by quantification of GFP-positive tumor cells after coculture (see **Figures 4A–E** for 9mer-C08 TCR-T cells and **Figures 4F–J** for 10mer-C08 TCR-T cells from patient CRI-5268, and **Supplementary Figures 3A–E** for 9mer-C08 TCR-T cells and **Supplementary Figures 3F–J** for 10mer-C08 TCR-T cells from patient CRI-5126, and **Supplementary Figures 3K–N** for 10mer-A11 TCR-T cells from patient CRI-5178). CKT manufactured TCR-T cells generally demonstrated more rapid killing of 2D and 3D cancer cell lines compared to IL-2 manufactured TCR-T cells (**Figures 4K–O**).

### Establishment of a clinical-scale TCR-T manufacturing process using the cytokine cocktail IL-2, IL-7, IL-15, and TGF-β

**Figure 5:**
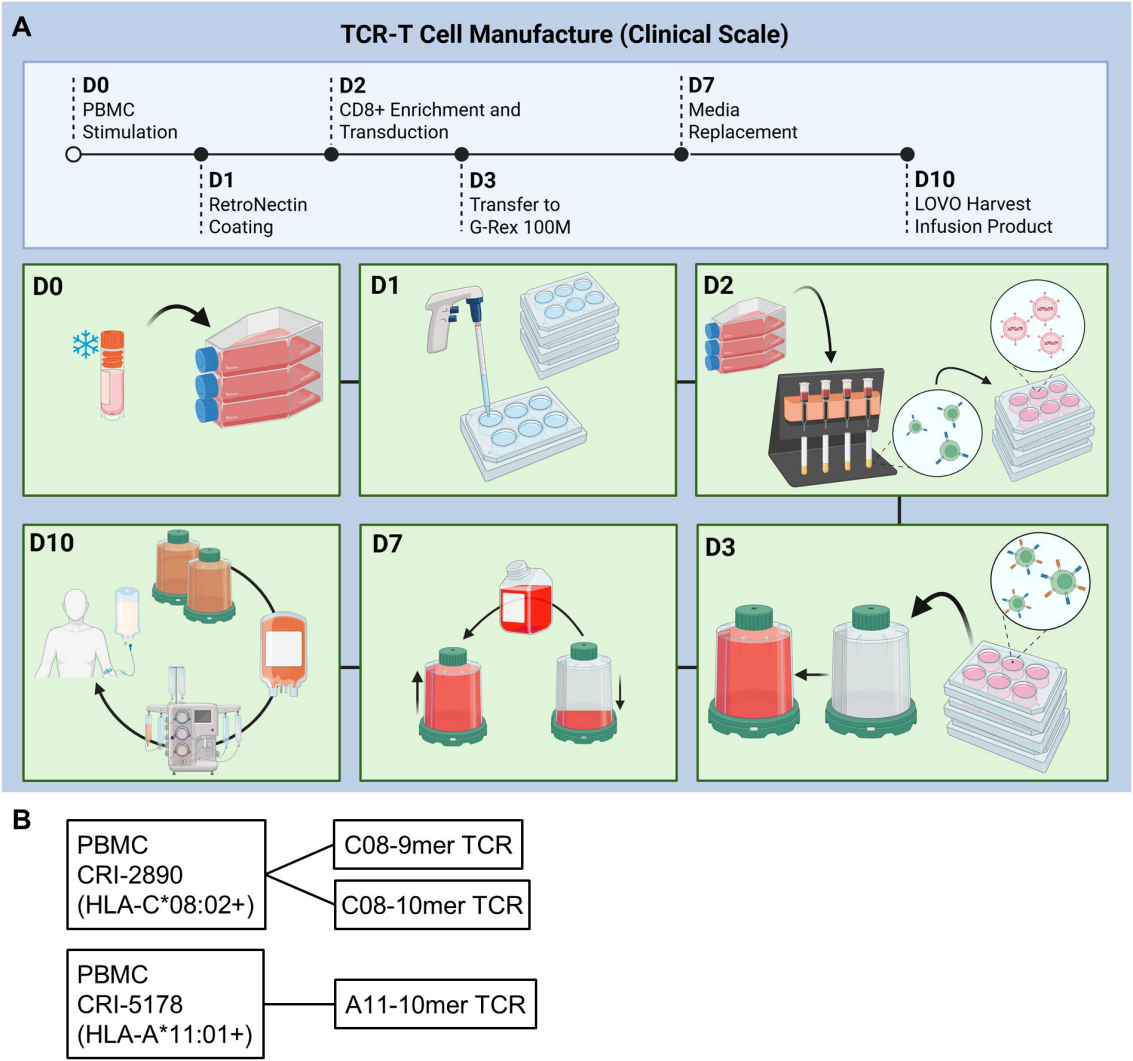
Clinical scale 10-day TCR-T manufacture process using CKT. **(A)** Schematic and **(B)** samples evaluated and TCRs used in the clinical-scale engineering runs. **(A)** was generated with BioRender.

**Figure 6:**
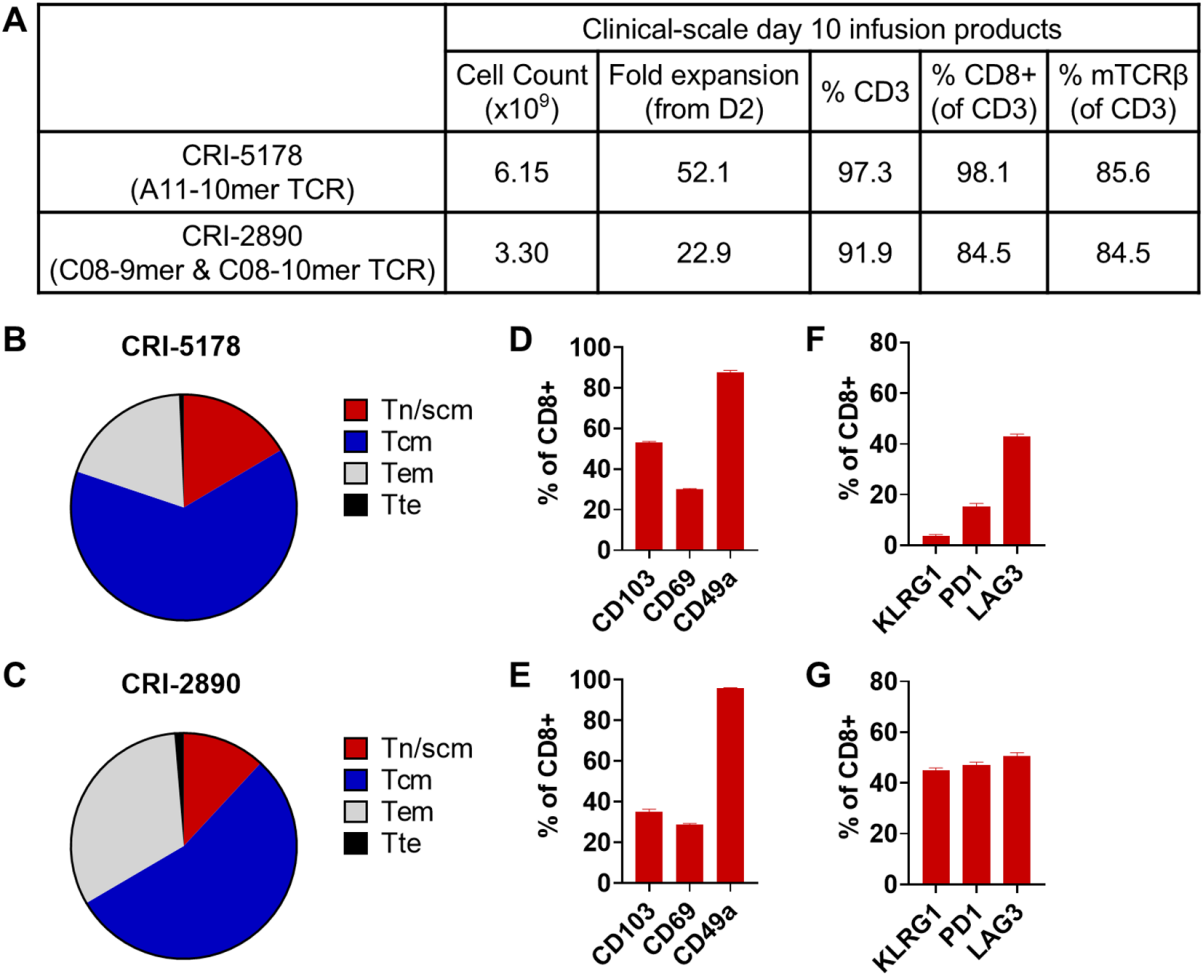
Cell number and phenotype of clinical-scale, 10-day CKT manufactured TCR-T cells. **(A)** Summary data for cell number (after LOVO harvest), fold expansion (from day 2 onwards), CD3 and CD8 frequencies, and transduction efficiency as determined by flow cytometric analysis of the mouse TCRβ constant region which is engineered into the TCRs. T-cell differentiation, tissue resident (Trm) and exhaustion/activation markers for TCR-T infusion products from CRI-5178 **(B, D, F)** and CRI-2890 **(C, E, G)** as determined by flow cytometric analysis. Tn/scm: CD45RO-CCR7+; Tcm: CD45RO+CCR7+; Tem: CD45RO+CCR7-; Tte: CD45RO-CCR7-.

**Figure 7:**
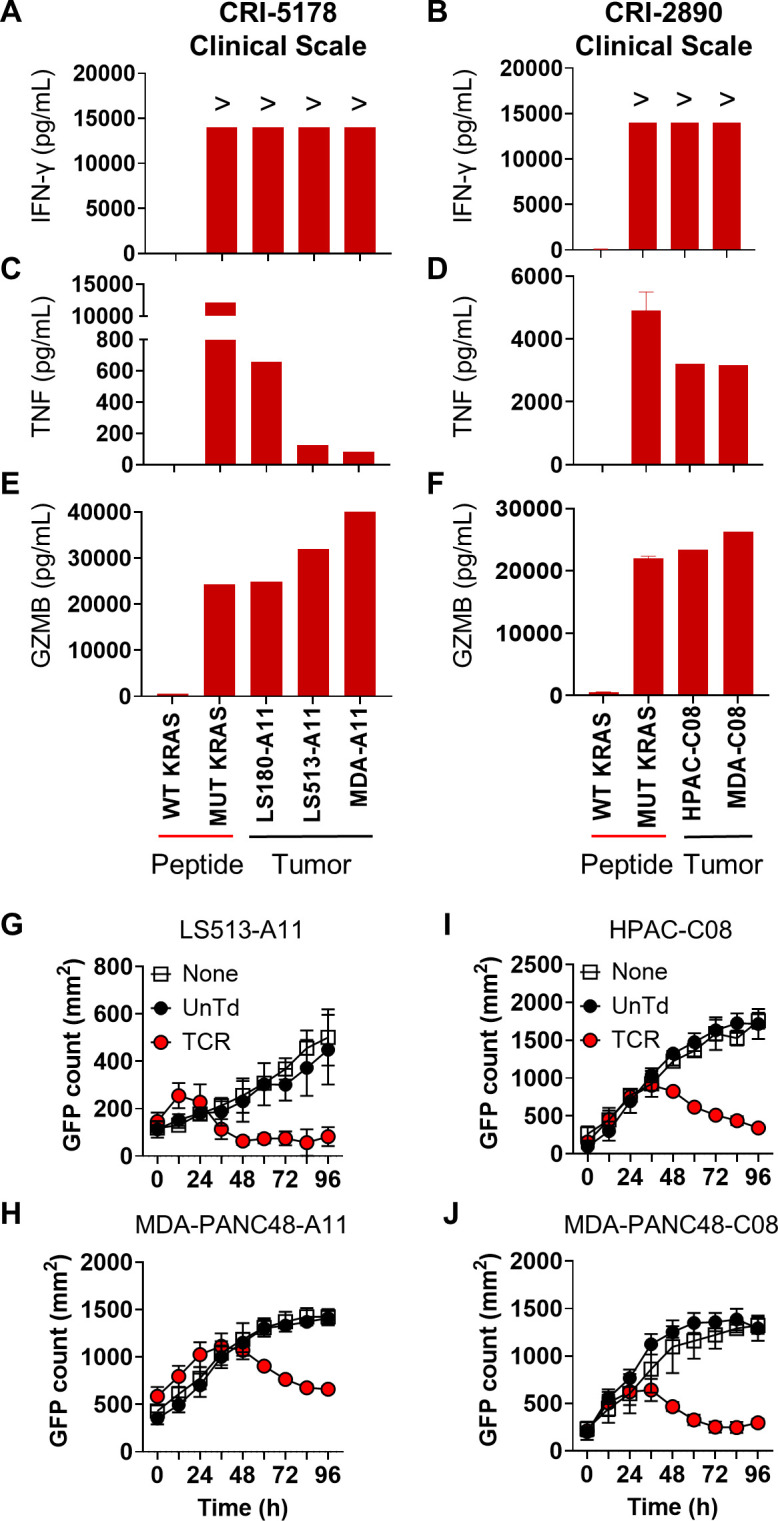
Effector function and antitumor activity of clinical-scale, 10-day CKT manufactured TCR-T cells. CRI-5178 10mer-A11 (left) or CRI-2890 9mer-C08 (right) TCR-T cells manufactured at clinical scale were cocultured overnight with the indicated KRAS G12D+ tumor cell lines expressing either HLA-A*11:01 (A11) or HLA-C*08:02 (C08) or autologous PBMC pulsed with 1 µg/mL of wild-type (WT) or mutated (MUT) KRAS peptide and **(A, B)** IFN-γ, **(C, D)** TNF, and **(E, F)** granzyme B (GZMB) was measured in the supernatants using the LegendPlex assay. **(G, H)** CRI-5178 10mer-A11 and **(I, J)** CRI-2890 9mer-C08 TCR-T were cocultured with the indicated KRAS G12D+ and A11 or C08 GFP-expressing tumor cell lines at an effector to target (E:T) ratio 1:2 in 2D culture and GFP was measured using the Cellcyte live cell imager. “>“ greater than assay detection limit. None, tumor cells alone; UnTd, untransduced T cells. LEGENDplex data were from one experiment with technical replicates. Coculture assays are representative of at least 2 independent experiments.

The favorable phenotype and robust antitumor activity observed with TCR-T cells produced in 10 days with CKT led us to develop a clinical-scale manufacturing protocol. This protocol (**Figure 5A**) was identical to our small-scale protocol (**Figure 1A**) except that reagents and materials were scaled up accordingly and the final infusion products were harvested with the LOVO Cell Processing System. To achieve clinically relevant TCR-T cell numbers, we stimulated 1.2 × 10^9^ PMBC and transduced about 1.44 x 10^8^ CD8-enriched T cells which were expanded in two G-Rex 100M flasks. We performed two engineering runs using PBMCs from patients with pancreatic cancer: CRI-5178, previously used in our small-scale studies, and CRI-2890, a new donor (**Figure 5B**). As seen in **Figure 6A**, the two CKT engineering runs with CRI-5178 and CRI-2890 yielded 3.30 and 6.15 x 10^9^ total cells after harvest, about 98% and 85% CD8 T cells, and 86% and 85% TCR transduction efficiency, respectively. Consistent with the small-scale studies, the infusion products were composed predominantly of Tn/scm and Tcm (**Figures 6B, C**), with expression of the Trm markers CD103, CD69, and CD49a (**Figures 6D, E**), and variable expression of KLRG1, PD-1, and LAG3 (**Figures 6F, G**). Moreover, stimulation with mutated KRAS peptides or coculture with KRAS G12D+ tumor cell lines expressing the appropriate HLA led to robust production of IFN-γ, TNF, and GZMB (**Figures 7A–F**) which was associated with *in vitro* tumor cell line killing as expected (**Figures 7G–J**). Both CKT infusion products from the engineering runs passed safety tests including vector copy number (VCN), replication competent retrovirus (RCR), endotoxin, mycoplasma, and sterility (**Supplementary Table 1**).

## Discussion

A major feature of ACT is the ability to manipulate manufacture conditions to endow T cells with desired phenotypic and functional attributes. Here, we developed a clinical-scale, 10-day manufacturing protocol utilizing a novel cytokine cocktail (CKT) containing IL-2, IL-7, IL-15, and TGF-β to generate TCR-T-cell products enriched in early memory and tissue-resident-like phenotypes and with enhanced *in vitro* effector function and antitumor ability. A large body of evidence exists demonstrating the superiority of early memory T cells over more differentiated T cells in antitumor immunity (18) and thus we utilized IL-7 and IL-15 for their established roles in promoting T-cell proliferation while preserving Tn/scm and Tcm phenotypes during cell manufacture (30, 32, 33). Although IL-2 is a known driver of terminal differentiation (37, 38), it was included in our cytokine cocktail because preclinical observations in one of our patient-derived PBMC samples indicated that IL-7 and IL-15 alone were insufficient to drive robust TCR-T cell expansion; the addition of IL-2 rescued this proliferative capacity (data not shown). Notably, despite the inclusion of IL-2, a significant fraction of early memory T cells was preserved in the final TCR-T cell products across all four patient samples (**Figures 2G**, **6B, C**). The final component of our CKT is TGF-β which is perhaps most well known as an immunosuppressive cytokine but has pleiotropic effects that are context dependent (39). Indeed, we incorporated TGF-β because of its critical role in generating Trm (40–44), which are positively correlated with patient survival in many tumor types as well as implicated in mediating responses to immunotherapy (25, 26).

The exact mechanisms of how our CKT-manufactured TCR-T cells mediate improved antitumor activity have not been fully elucidated but this and prior studies may provide some insight. In the setting of preclinical ACT studies, the use of TGF-β to generate more effective human T cells was first reported in the setting of tumor-infiltrating lymphocytes (TIL) (45). In this study, addition of TGF-β at the beginning of the rapid expansion protocol favored growth of CD8 TIL over CD4 TIL which led to enrichment of melanoma antigen (MART1)-reactive CD8+ TIL and increased antitumor activity *in vitro*. In another study (46), TGF-β exposure during cell manufacture programmed T cells into a Tcm state with high polyfunctionality and proliferative potential which was associated with improved control of tumor xenografts by BCMA-targeted CAR-T cells. More recently, Jung et al. incorporated TGF-β during CAR-T cell manufacture which drove chromatin and transcriptional changes that promoted both stem-like and Trm phenotypes leading to improved tumor clearance via increased effector cytokine production, tumor infiltration, and *in vivo* persistence (27). Our CKT manufactured TCR-T cells similarly demonstrated an increase in early memory/Trm phenotypes and effector cytokine production. However, unlike Jung et al. who observed a decrease in cytotoxicity *in vitro* when TGF-β was added to IL-2 during CAR-T cell manufacture, we found that CKT increased granzyme B production and cytotoxicity relative to IL-2 manufactured TCR-T (**Figures 3K, J, L**; **Figure 4**; **Supplementary Figure 3**). Furthermore, while Jung et al. observed decreased *KLF2* gene expression, which is consistent with a Trm phenotype, we did not observe this trend at the protein level in CKT-manufactured TCR-T cells (data not shown). This could be a consequence of CKT containing IL-7 and IL-15 which are known to promote expression of KLF2 (47, 48). Beyond cytokine signaling, these discrepancies also could be influenced by our use of patient-derived PBMCs and specific enrichment for CD8+ T cells, which may create a distinct epigenetic and metabolic starting point compared to the healthy donor PBMCs and mixed CD4/CD8 products used by Jung et al. Additional transcriptomic, epigenetic, and proteomic studies on CKT and IL-2 manufactured TCR-T cells would shed further light into the mechanisms responsible for these improved antitumor attributes. Notably, the effects of TGF-β on enhancing T-cell function do not appear restricted to conventional αβ T cells since γδ (Vγ9Vδ2) T cells expanded with IL-2 and TGF-β also demonstrate enhanced effector cytokine production, cytotoxicity, and antitumor activity compared to those expanded with IL-2 (49, 50).

While our current study demonstrated that TCR-T cells generated in 10 days with CKT displayed improved phenotype and function compared to those produced with IL-2, we further evaluated these cells against our previous manufacturing standard. This original protocol was a two-phase process involving initial PBMC stimulation and transduction, followed by a rapid expansion protocol (REP) to generate the final infusion product as described in Leidner et al. (16). Notably, we had previously manufactured a clinical-scale infusion product for patient CRI-2890 using this IL-2 based two-step method (V1; **Supplementary Figure 4A**), which allowed for a direct comparison between the two manufacturing approaches. In contrast to the CRI-2890 CKT infusion product (**Figures 6C, E, G**), the V1 product consisted almost entirely of Tem and Tte cells that lacked CD103 and CD69 expression and showed elevated KLRG1, though they maintained lower levels of PD-1 and LAG3 (**Supplementary Figures 4B–D**). Furthermore, we observed increased tumor cell killing by the CKT manufactured infusion product compared to the V1 infusion product of patient CRI-2890 (**Supplementary Figures 4E, F**). Taken together, these data indicate that TCR-T cells generated using CKT exhibit a more favorable phenotypic and functional profile than those produced by two distinct IL-2-based protocols.

Most TCR-T cell products evaluated in clinical trials to date employ HLA-I-restricted TCRs and are generated from bulk PBMCs without subset enrichment (1–7, 9–16, 51). Although this approach simplifies manufacturing, the resulting variability in CD4+ and CD8+ T-cell frequencies may impact efficacy. Specifically, HLA-I-restricted TCRs typically exhibit higher functional avidity in CD8+ T cells than in CD4+ T cells due to CD8-coreceptor-mediated enhancement of TCR engagement with HLA-I (52, 53). Consequently, infusion products skewed toward CD4+ T cells may exhibit suboptimal therapeutic activity. Indeed, this is supported by our preclinical work, where CD8-enriched TCR-T cells from two of three patient PBMC samples displayed improved tumor cell killing *in vitro* compared to bulk TCR-T cells (**Supplementary Figure 5**). Notably, recent TCR-T clinical trials targeting the tumor antigens MAGE-A4 (10) and PRAME (12) reported a median of ~ 50% CD4+ T cells in the final products, with some reaching as high as ~ 80%. To mitigate this inherent variability and ensure a defined and potentially more potent product, we incorporated a CD4+ cell depletion step to enrich CD8+ T cells during manufacturing. Another strategy to address this includes genetically engineering the bulk T-cell population to express CD8α (54) or CD8α/β co-receptors (52), an approach being explored in TCR-T clinical trials targeting MAGE-A4 (e.g., NCT04044859), PRAME (NCT03686124), and KRAS G12D (NCT06218914; biological product AZD0240).

Minimizing vein-to-vein time is critical for the clinical deployment of ex vivo cell therapies, particularly for patients with rapidly progressing disease. Manufacturing time for most TCR-T products typically ranges from seven days to a month, often with additional time for release testing, and thus our 10-day manufacturing process with fresh (non-cryopreserved) product infusion compares favorably. Infusion with fresh product is possible because our cleanroom facility is located at the hospital where patients would receive treatment. This point-of-care manufacturing model enables a shorter vein-to-vein time and mitigates the potential negative impacts of cryopreservation and thawing on the cell product.

Our study has several limitations. While our CKT-manufacturing process generated consistent trends on the phenotype and function of TCR-T cells across four patient samples, the biological diversity of patient-derived PBMCs will inevitably impact the final phenotype, yield, function, and ultimately, the antitumor potential of individual infusion products. Nonetheless, the use of patient-derived samples in this study represents an improvement over the healthy donor PBMCs commonly used in preclinical studies. When advancing an experimental cell therapy product toward larger-scale pivotal studies, a transition to simple, robust, automated, and closed systems is desirable. Our current 10-day manufacturing process involves significant manual manipulation, using tissue culture flasks during PBMC stimulation, columns for CD8 enrichment, and 6-well plates for transduction. Although this process is performed under GMP conditions and achieves therapeutic doses with high transduction efficiency in 10 days, further process development is required to implement automation in a closed system. The G-Rex technology used in this study supports closed-system processes, as do other platforms such as the CliniMACS Prodigy, which has been employed to develop TCR-T cells in an automated, closed, and GMP-compliant process (55, 56). Finally, our study is limited to *in vitro* characterization, and therefore it remains to be determined whether the desirable attributes imprinted by CKT on the TCR-T cells translate to improved control of tumors *in vivo*.

In conclusion, we have developed a 10-day, clinical-scale manufacturing protocol that utilizes a novel cytokine cocktail consisting of IL-2, IL-7, IL-15 and TGF-β to generate TCR-T cells with early memory and tissue-resident-like phenotypes and robust *in vitro* antitumor activity. We plan to evaluate the safety and efficacy of mutant KRAS-targeting TCR-T cells generated with this protocol in an upcoming clinical trial.

## Data Availability

The raw data supporting the conclusions of this article will be made available by the authors, without undue reservation.
