# Supplementary material for: Clinical-scale 10-day TCR-T cell manufacturing using IL-2/7/15 and TGF-β promotes early memory and tissue-resident-like phenotypes and robust antitumor activity in vitro
Source: Front Immunol. 2026 May 15;17:1847411. doi: 10.3389/fimmu.2026.1847411 (PMC13218850; doi:10.3389/fimmu.2026.1847411)
Supplement: Supplementary file 1 [file SupplementaryFile1.pdf]

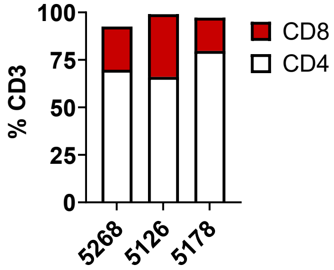

Supplementary Figure 1. Proportion of CD8 and CD4 T cells in patient PBMC prior to stimulation. PBMCs from CRI-5268, CRI-5126, and CRI-5178 were stained with fluorochrome-conjugated antibodies followed by flow cytometric analysis. Data are gated on live CD3+ cells.

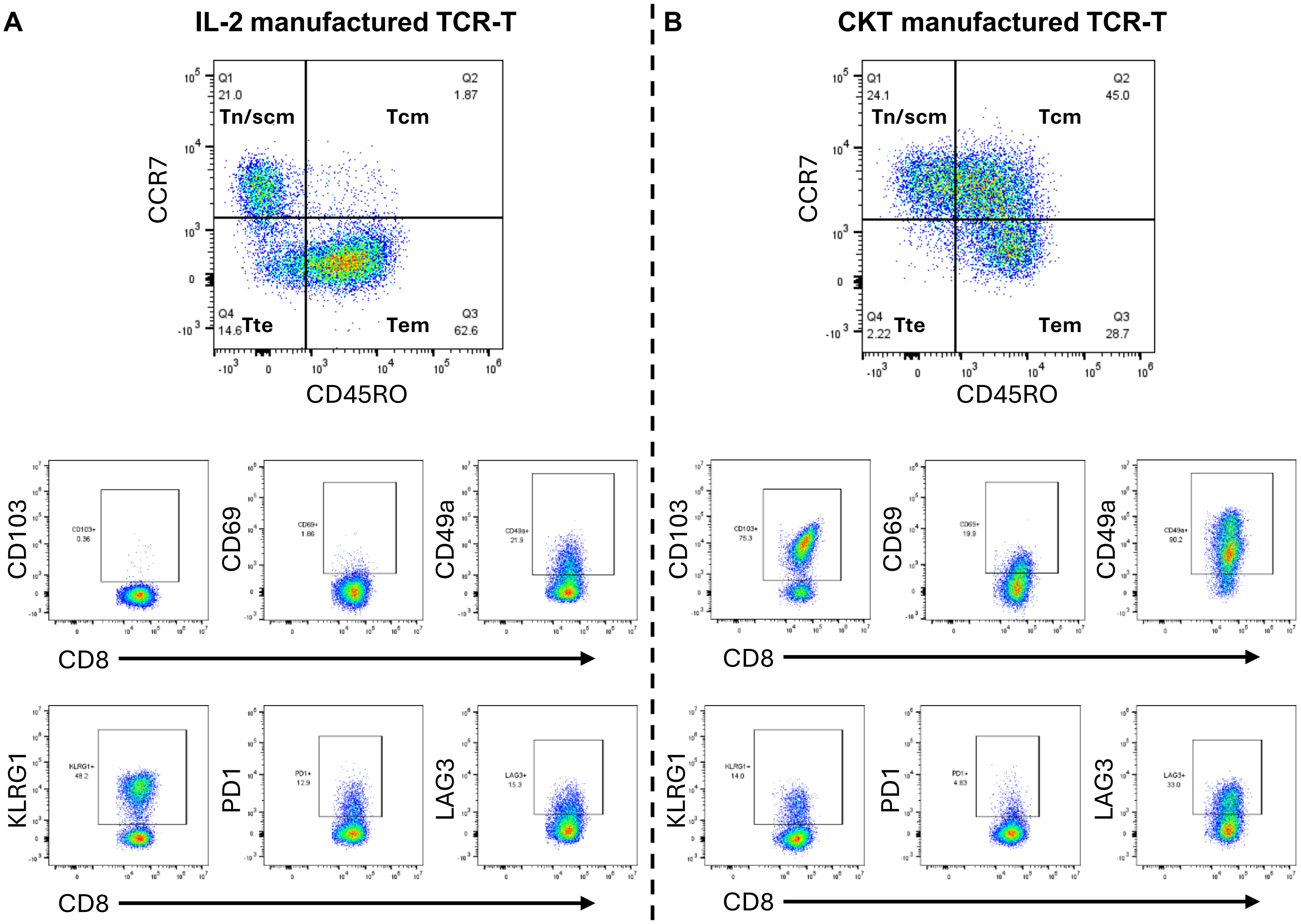

Supplementary Figure 2. Example flow cytometry plots of phenotypic markers expressed on TCR-T cells after manufacture with IL-2 or cytokine cocktail for 10 days. Differentiation (top), tissue resident (middle), and activation/exhaustion (bottom) markers for patient CRI-5268 manufactured with either (**A**) IL-2 or (**B**) CKT and engineered to express the C08-9mer TCR. Data were gated on live CD3 and CD8 lymphocytes.

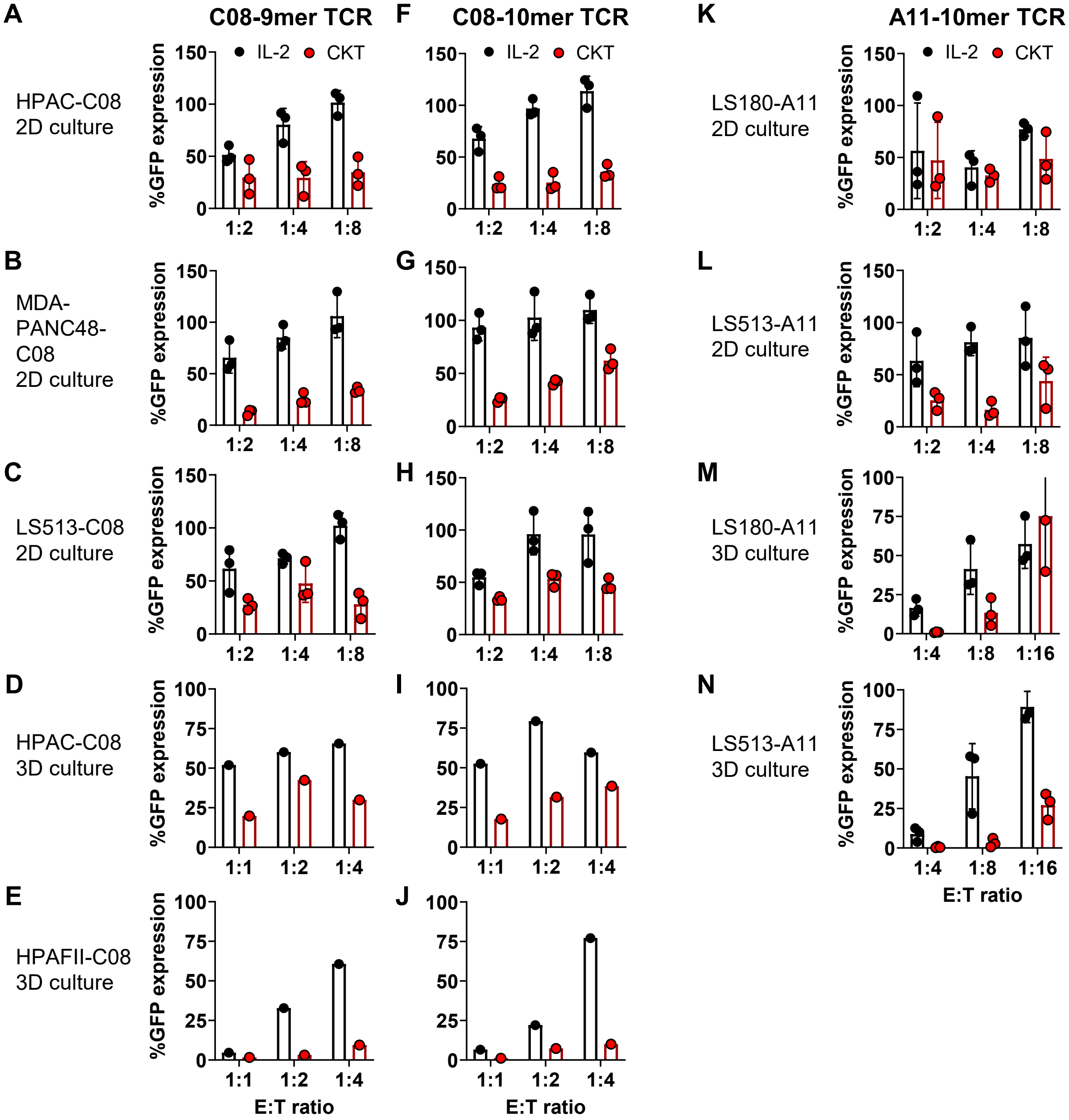

Supplementary Figure 3. Tumor cell killing by IL-2 or CKT manufactured TCR-T cells from patients CRI-5126 and CRI-5178 in 2D and 3D coculture. (**A-E**) 9mer-C08 TCR-T cells manufactured with IL-2 (black) or CKT (red) from CRI-5126 were cocultured with the indicated KRAS G12D+ and HLA-C*08:02+ GFP-expressing tumor cell lines at various effector to target (E:T) ratios in (**A-C**) 2D or (**D-E**) 3D spheroid culture and GFP was quantitated at 72 h. (**F-J**) Same as (A-E) except with 10mer-C08 TCR-T cells. (**K-L**) 10mer-A11 TCR-T cells manufactured with IL-2 (black) or CKT (red) from CRI-5178 were cocultured with the indicated KRAS G12D+ and HLA-A*11:01+ GFP-expressing tumor cell lines at various effector to target (E:T) ratios in (**K-L**) 2D or (**M-N**) 3D spheroid culture and GFP was quantitated at 72 h. 100% GFP expression indicates the % GFP+ tumor cells in coculture wells containing control untransduced T cells manufactured with IL-2 or CKT. Coculture assays are representative of at least 2 independent experiments.

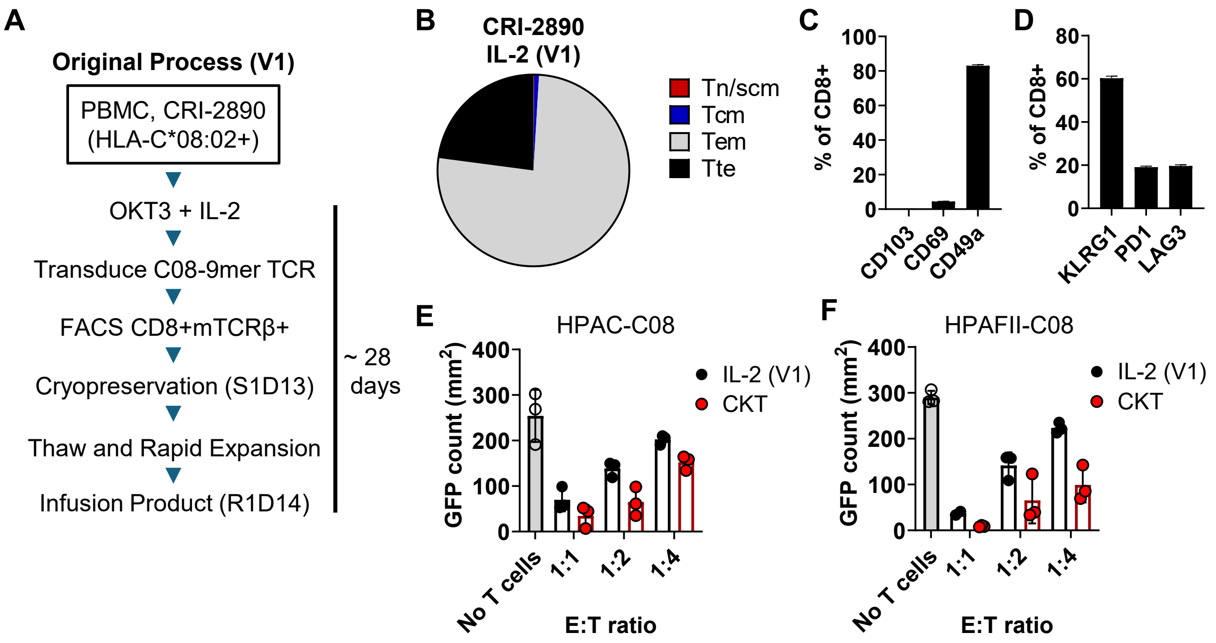

Supplementary Figure 4. Phenotypic and functional characterization of TCR-T cell infusion product manufactured using our original process with IL-2 (V1) from patient CRI-2890 PBMC. (**A**) Schematic of the process used to generate the TCR-T cell infusion product from patient CRI-2890 PBMC using our original process (V1). (**B**) T-cell differentiation, (**C**) tissue resident (Trm), and (**D**) exhaustion/activation markers as determined by flow cytometric analysis. (**E-F**) CRI-2890 9mer-C08 TCR-T cells manufactured at clinical scale with either the new 10-day process with CKT (red) or with the original ~ 28 day process (V1) using IL-2 (black) were cocultured with the indicated KRAS G12D+ and HLA-C*08:02+ GFP-expressing tumor cell lines at various effector to target (E:T) ratios in 2D culture and GFP was quantitated at 72 h with the Cellcyte live cell imager. FACS, fluorescence-activated cell sorting; S1D13, first stimulation, day 13; R1D14, rapid expansion protocol, day 14. Flow cytometry data were gated on CD3+CD8+ T cells. Tn/scm: CD45RO-CCR7+; Tcm: CD45RO+CCR7+; Tem: CD45RO+CCR7-; Tte: CD45RO-CCR7-. UnTd, untransduced T cells. Coculture assays are representative of at least 2 independent experiments.

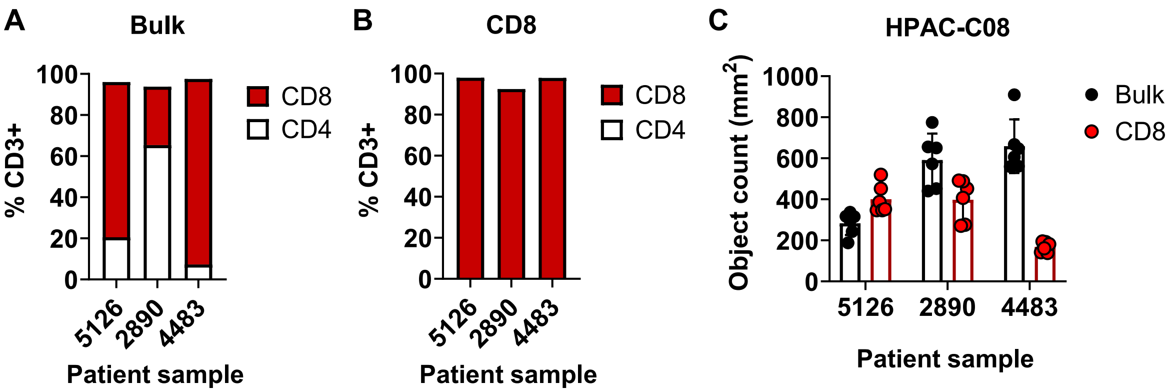

Supplementary Figure 5. In vitro cytotoxicity of bulk versus CD8-enriched TCR-T cells manufactured in 10 days with CKT. Frequency of CD4 and CD8 T cells within (**A**) bulk or (**B**) CD8-enriched TCR-T cells derived from patients CRI-5126, CRI-2890, and CRI-4483 used in (**C**) the in vitro coculture with the KRAS G12D+ and HLA-C*08:02+ GFP-expressing tumor cell line HPAC-C08 at an effector to target (E:T) ratio 1:8. GFP was quantitated at 96 h with the Cellcyte live cell imager. Flow cytometry data were gated on live CD3+ cells. Note, in this experiment, CD8-enrichment, via CD4 depletion, was performed at the end of the 10-day manufacture process. The experiment was performed once.

Supplementary Table 1. TCR-T engineering runs safety testing summary

	Clinical-scale day 10 infusion products				
	Vector copy number per cell, MSGV QPCR	RCR, RD114 QPCR trend over time (D3/7/10)	Endotoxin, Endosafe nexgen-PTS (EU/mL)	Mycoplasma, MycoTOOL RT-PCR	Sterility, Bac-T
CRI-5178 (A11-10mer TCR)	2.11	Down	<0.108	≤ 10 CFU/mL	Negative
CRI-2890 (C08-9mer & C08-10mer TCR)	2.46	Down	< 0.100	≤ 10 CFU/mL	Negative
